# Reduced performance of a PVC-coated Biogents Sentinel prototype in comparison to the original Biogents Sentinel for monitoring the Asian tiger mosquito, *Aedes albopictus*, in temperate North America

**DOI:** 10.1371/journal.pone.0172963

**Published:** 2017-03-20

**Authors:** Isik Unlu, Ary Faraji, Michael Morganti, Randy Vaeth, Karen Akaratovic, Jay Kiser, Charles Abadam, Dan Kline

**Affiliations:** 1 Center for Vector Biology, Rutgers University, New Brunswick, New Jersey, United States of America; 2 Mercer County Mosquito Control, West Trenton, New Jersey, United States of America; 3 Salt Lake City Mosquito Abatement District, Salt Lake City, Utah, United States of America; 4 East Baton Rouge Parish Mosquito Abatement and Rodent Control, Baton Rouge, Louisiana, United States of America; 5 City of Suffolk Mosquito Control, Suffolk, Virginia, United States of America; 6 USDA-ARS, CMAVE, Gainesville, Florida, United States of America; University of Thessaly School of Agricultural Sciences, GREECE

## Abstract

*Aedes albopictus* is a major nuisance pest and also a public health concern because of the role it plays in the transmission of arboviruses. There is a continuing demand for effective surveillance tools for this species. The first generation of Biogents Sentinel (BGS1) traps have proven to be an effective tool for surveillance of *Ae*. *albopictus* throughout its range, however, some defects in construction led to the eventual development of the next generation. We compared the performance of the new generation prototype trap (BGS2P) to the original. Studies were conducted in suburban and urban areas in Florida, Louisiana, New Jersey, and Virginia, USA in the summer of 2014 (July-October). BGS1 traps collected significantly more *Ae*. *albopictus* when compared to the BGS2P with or without CO_2_ in all locations (*P*<0.05). When a white cloth was wrapped around the BGS2P traps, efficiency did not change in Louisiana, New Jersey, and Virginia; however, numbers of adult *Ae*. *albopictus* collected from the BGS2P and the BGS1 were significantly different based on lure type (*P*< 0.0001). Results from Florida showed that BGS1with the BG lure and CO_2_ collected significantly higher adult numbers compared to BGS2P with a three component cartridge lure and CO_2_ (*P*< 0.0001). Overall, our results indicate that despite improvements in construction and durability of the BGS2P, this newer trap type did not increase the capture rates of *Ae*. *albopictus* in North America. Biogents modified BGS2P based on the data collected from the current study and updated as Biogents Sentinel 2 is now commercially available and its efficacy in comparison to the original will require further study.

## Introduction

Following the development of the New Jersey light trap (NJLT) in 1932, adult mosquito surveillance became more standardized and efficient [1]. The NJLT soon became the gold standard for monitoring adult mosquito populations and it has since been used consistently by mosquito control programs globally [2]. In the 1960’s the Centers for Disease Control (CDC) miniature light trap, using light and CO_2_, was developed as a highly efficient trap for collecting host-seeking adult mosquitoes [3].Currently, the standard NJLT and the CDC miniature light traps are widely used for arbovirus surveillance in the United States and abroad [4]. However, since these traps mostly target host-seeking nulliparous mosquitoes, they are unlikely to collect specimens that have been exposed to arboviruses [5]. Reiter (1983) introduced a portable, battery powered gravid trap (GT) and demonstrated that at least 90% of the mosquitoes collected with this trap were gravid [5]. Unfortunately, diurnal mosquito species such as *Aedes* (*Stegomyia*) *albopictus* (Skuse) do not respond well to light or gravid traps [6,7]. Possible reasons might be that light traps are usually placed with their openings 1.5 m above the ground and are operated at night using a light source as an attractant. *Aedes albopictus* is primarily diurnal, usually host-seeking near ground level, [8] and is not attracted to light sources. Even though gravid traps are more promising than light traps, they are still not effective trapping tools for *Ae*. *albopictus* surveillance because of the low catch counts [7].

The Biogents Sentinel (BGS) trap (Biogents AG, Regensburg, Germany) was originally designed to capture *Aedes* (*Stegomyia*) *aegypti* (L.), but it has also proven efficient for *Ae*. *albopictus* [9].The BGS trap uses a combination of visual cues, chemical attractants, and convection currents to capture host-seeking mosquitoes. Studies have shown that a BGS trap without chemical attractants may still capture *Aedes* mosquitoes, which indicates that visual cues also play a key role in attracting these mosquitoes [10]. The BGS traps may be operated with three main types of chemical attractants: an octenol (1-octen-3-ol) lure sachet (AgriSense, Pontypridd, United Kingdom), a mesh BG lure containing ammonia, lactic acid, and fatty acids (Biogents AG), or a BG cartridge lure (Biogents AG). When a BGS trap is operating, the plume of the chemical attractant is forced up and outward from the trap through the white gauze cover. Mostly host-seeking mosquitoes are attracted towards the scent and then suctioned into a collection net via a fan located at the bottom of the trap opening [11].

The BGS trap has made it possible to examine the temporal and spatial population dynamics of *Ae*. *albopictus* and evaluate the effectiveness of control interventions [12,13]. However, the construction of BGS1 trap has posed challenges for researchers and public health stewards [14]. Furthermore, maintenance of the traps to continue season-long surveillance requires substantial budget and work hours [14]. This study describes the new prototype with an improved design meant to address previous defects (related with the fan and wiring), and compares the efficacy between the new BGS trap prototype (BGS2P) and the original BGS1. To increase the durability of the trap, polyvinyl chloride (PVC) coated fabric was used for the prototype instead of polyethylene (PE) non-woven fabric.

## Materials and methods

### Ethics statement

No specific permits were required for the described field studies, which were developed with business or home owners assent by professional county mosquito control personnel. These studies did not involve endangered or protected species.

### Study site description

Our studies were conducted at one site in Florida, two sites in Louisiana, one site in New Jersey, and one site in Virginia, USA. All study sites were historically known to support high populations of *Ae*. *albopictus*. The Florida site was a residential backyard (29° 43’ N, 82° 23’) located within the city limits of Gainesville. The dominant vegetation consisted of a mixture of longleaf pine (*Pinus palustris* Mill) and hardwood trees, mainly live oak (*Quercus virginiana* Mill) and water oak (*Quercus nigra* L.) with moderate amounts of shrubs, predominantly azalea (*Rhondodendron* spp.) and camellia (*Camellia*s spp.). The Louisiana sites were approximately 1,080m^2^ each consisting of residential blocks approximately 2.8 km apart in typical urban areas in Baton Rouge (30° 45´ N, 91° 14´ W). One site had more vegetation, but both were dominated by oak trees (*Q*. *viginiana*). The New Jersey site was located in Trenton (40° 14´ N, 74´ 44´ W) near an abandoned industrial site adjacent to a highway, surrounded by industrial businesses. The site was 8,100m^2^ dominated by catalpa trees (*Catalpa speciose* Warder. ex Barney) which provided shade for trap placement[15]. This area was a common dumping site used by locals to discard tires and other trash. The Virginia site was set within a wooded area approximately 25,000 m^2^ in downtown Suffolk (36° 42´ N, 76° 35´ W), surrounded by residential and industrial areas as well as wooded lots of varying size which contained hundreds of tires in piles. The site is located 1,500m south of the center of downtown and 2,400m west of the Great Dismal Swamp National Wildlife Refuge.

### BGS trapping protocols

**Fig 1:**
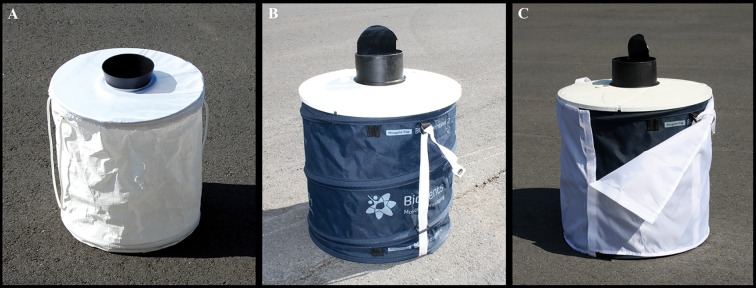
The three variations of the Biogents Sentinel trap. (A) Original BGS1. (B) PVC-coated BGS2 prototype (BGS2P). (C) PVC-coated BGS2P with optional white cover.

We compared the original BGS trap (BGS1) with the newly developed prototype (BGS2P). The new BGS2P has been designed to be more user-friendly and sturdy compared to its predecessor. The BGS2P remains collapsible and uses a self-supporting design, eliminating the need for mounting poles. The new canvas PVC-coated material covering the trap body is stronger than the previous version’s PE non-woven fabric material and the body of the trap is navy blue instead of white (Fig 1). The housing cover has a hard plastic frame which is much stronger, but still ventilated enough to allow the air plume to disperse the scent of an attractant. The collection pipe has also been changed and now includes an air-actuated flap, preventing the loss of captured specimens in the event of a power failure. The placement and construction of the BG lure has changed as well. The lure is designed as a cartridge, not a pouch, and fits directly into the housing cover. Although the trapping period varied slightly among states, the majority of the trap deployments took place between June and September, the peak activity time for *Ae*. *albopictus* in the study areas.

### Experiment 1: Comparisons between BGS1 and BGS2P

Experiment 1 was conducted in Louisiana and New Jersey with the following trap configurations: 1) BGS1 with no lure or CO_2_, 2) BGS1 baited with CO_2_, 3) BGS2P with no lure or CO_2_, 4) BGS2P baited with CO_2_. Each trap configuration was rotated through four predetermined trapping locations located a minimum distance of 20 m apart. In Louisiana and New Jersey, traps were given unique identification numbers and randomly assigned to a trapping location each week using a random number generator (MS Office Excel^™^ 2007; Microsoft Corporation, Redmond, WA, USA). After the initial random assignment, traps were rotated counter-clockwise by one position daily. This protocol for random trap assignment and rotation was used throughout the study for all experiments in Louisiana and New Jersey between July and September in 2014. Two kilograms of dry ice pellets were wrapped in newspaper and placed inside the trap as the CO_2_ source. A total of 16 trapping nights in Louisiana (n = 4 traps, 8 July to 1 August) and 18 trapping nights (n = 8 traps, 18 July to 15 August) in New Jersey were conducted. Sampling was performed continuously for a 24 h period, with each session starting between 10:00 am and 11:00 am.

### Experiment 2: Comparisons between various lures using BGS2P

One study was conducted in New Jersey with the following trap configurations: 1) BGS2P with no lure, 2) BGS2P baited with mesh BG lure, 3) BGS2P baited with new 4-compound BG cartridge lure (4C; lactic acid, hexanoic acid, ammonia, and octenol). No CO_2_ source was used in this experiment. Three traps were used for each configuration, with a total of 17 trapping nights (n = 9 traps; 12 September to 16 October).

Another study conducted in Virginia tested the effectiveness of four different lure types for collecting *Ae*. *albopictus* adults with BGS2P, which were all covered with the provided white cloth and supplied with CO_2_. The CO_2_ was provided by gas cylinders using a dispersal rate of 500 cc/min. Traps were randomized using the same protocol as New Jersey, described in the previous section. This experiment was done with the following trap configurations: 1) BGS2P with no lure 2) BGS2P with mesh BG lure, 3) BGS2P with cartridge 3-compound (3C; lactic acid, hexanoic acid, and ammonia) lure, 4) BGS2P with the 4C, 5) BGS2P with octenol-only BG cartridge lure (1C). Following a 5 x 3 Latin Square design, traps were placed approximately 20 m apart, set between 7:00 am and 7:15 am, and picked up between 6:45 am and 7:00 am. One trap was used for each configuration. This experiment consisted of three replicates totaling 15 trapping nights (n = 5 traps; 24 July to 16 August).

### Experiment 3: Addition of white cloths to BGS2P

To determine if the darker trap body had any effect on *Ae*. *albopictus* collections, we wrapped the BGS2P traps with the optional white cloth. A previous study reported that modifying the BGS1 trap by wrapping a black cloth around the trap body led to an increase in the number of *Ae*. *aegypti* adults that were captured[16]. This additional white cloth experiment was conducted in Louisiana and New Jersey using no CO_2_ in either experiment. In Louisiana, trap configurations were as follows: 1) BGS2P baited with 4C lure, and 2) BGS2P with white cloth baited with 4C lure. The experiment consisted of a total of 24 trapping nights (n = 4 traps) and was conducted at two different sites (12 trapping sessions per site) between 16 July and 2 September. New Jersey used a modified trapping protocol based on the number of available traps: 1) BGS2P with no lure, 2) BGS2P baited with 4C lure, 3) BGS2P with white cloth and no lure, and 4) BGS2P with white cloth baited with 4C lure.(n = 8 traps;19 August to 11 September). Virginia used mesh BG lures and CO_2_ for each trap configuration: 1) BGS1, 2) BGS2P, and 3) BGS2P white cloth. The CO_2_ was provided by gas cylinders and randomized as described in the first Virginia study. Following a 3 x 6 Latin Square design, traps were placed approximately 20 m apart, set between 7:00 am and 7:15 am, and picked up between 6:45 am and 7:00 am. One trap was used for each configuration. This experiment consisted of six replicates for a total of 18 trapping nights (n = 3; 25 June to 20 July).

### Experiment 4: Comparisons between various lures using BGS1 and BGS2P

Three experiments were conducted in Florida comparing BGS1 and BGS2P. The first used the following configurations: 1) BGS1 with no lure, 2) BGS2P with no lure. Second configurations as follows:1) BGS1 with mesh BG lure, 2) BGS2P with mesh BG lure. The third was configured as follows: 1) BGS1 with mesh BG lure and octenol, and2) BGS2P with mesh BG lure and octenol. One trap was used for each configuration for three nights (n = 6 traps; 13 to 25 September).

We used the BioSensory octenol lure for this study (BioSensory, Inc., Putnam, CT, USA). The BioSensory lure consisted of 3.75 g of a 50:50 R:S racemic blend of octenol formulated in 12 g of a patented blend of waxes, which slowly released octenol from a patented plastic dispenser.

### Data analysis

The total number of *Ae*. *albopictus* numbers from each state were analyzed separately for this study, because the experimental set up was not consistent among states. Adult *Ae*. *albopictus* counts (total number of females and males) from BGS1 and BGS2P traps were compared using negative binomial regression with a log link (PROC GENMOD, SAS version 9.4 for Windows) for all experiments except for data from New Jersey (Experiment 1), and Florida (Experiment 4). Overdispersion in the New Jersey model was not adequately accounted for by the negative binomial distribution, therefore a quasi-Poisson model, scaled using the Pearson chi-squared statistic divided by the degrees of freedom, was used instead. For all models, the total number of adults was regressed against time and trap type. Time was modeled as a continuous linear covariate for all comparisons; however, in Experiment 1, we used piecewise linear regression with a knot at day 22 to account for curvature at the end of the trapping period. All pairwise comparisons were examined using Holm’s correction to control for multiplicity. The Florida data were analyzed using chi-squared or exact chi-squared tests (PROC FREQ, SAS version 9.4 for Windows).

## Results

### Experiment 1: Comparisons between BGS1 and BGS2P

A total of 137 *Ae*. *albopictus* adults (90 females and 47 males) were collected using BGS1 and BGS2P in Louisiana. There was no significant interaction between trap type and date. Trap performance did not improve when baited with CO_2_ for either trap type. However, the number of adult *Ae*. *albopictus* collected from BGS1 were always significantly higher than from BGS2P (*P* < 0.0001), regardless of whether the traps contained a CO_2_ source or not (Table 1).

**Table 1:** Comparison of the number of adult *Ae*. *albopictus* collected using BGS1 and BGS2P traps with and without CO_2_ in New Jersey and Louisiana, USA.

Location	Trap type	Lure type	No. of trap nights	LS means of *Ae*. *albopictus* †	95% CI
**New Jersey**	BGS1	**No CO**_**2**_	66	8.1^a^	6.3–10.4
		CO_2_	66	17.0^b^	14.3–20.2
	BGS2P	No CO_2_	65	3.2^c^	2.1–4.8
		CO_2_	64	7.6^a^	5.9–9.9
**Louisiana**	BGS2P	No CO_2_	16	2.6^ab^	1.5–4.5
		CO_2_	18	3.7^a^	2.2–6.0
	BGS2P	No CO_2_	13	1.2^ab^	0.6–2.5
		CO_2_	13	1.1^b^	0.5–2.2

^†^ Values within trap types in each state that share the same superscript are not significantly different by the Holm’s test (*P*> 0.05).

In New Jersey, 2,458 *Ae*. *albopictus* adults (1,454 females and 1,004 males) were collected from all trap types. BGS1 and BGS2P baited with CO_2_ had higher catches (females and males combined) than those without (BGS1: Z = 4.82, *P*< 0.0001; BGS2P: Z = 3.58, *P* = 0.0007). Furthermore, BGS1 traps with CO_2_ outperformed BGS2P traps without CO_2_ (Z = 3.58, *P* = 0.0003) and BGS2P traps with CO_2_ (Z = 5.02, *P*<0.0001); however, there was no significant difference between trap catches from BGS1without CO_2_ and BGS2P with CO_2._

### Experiment 2: Comparisons between various lures using BGS2P

A total of 695*Ae*.*albopictus* adults (499 females and 196 males) were collected using BGS2P with and without BG mesh lure and 4C lure in New Jersey. Trap performance did not improve with the addition of either lure (Table 2).There were no significant differences in the number of adult *Ae*. *albopictus* collected using either of the two lures or no lure at all.

**Table 2:** Comparison of the number of adult *Ae*. *albopictus* collected using BGS2P traps with and without white cloth using various lures in New Jersey and Virginia, USA.

Location	Trap type	Lure type	No. of trap nights	LS Mean no. of *Ae*. *albopictus* †	95% CI
**New Jersey**	BGS2P	No lure	50*	4.8	3.7–6.2
		Mesh BG lure	51	4.5	3.5–5.9
		4C	50*	4.4	3.3–5.7
**Virginia**	BGS2P with white cloth	No lure	15	12.8^a^	9.3–17.7
		1C	15	23.6^b^	17.4–32.1
		Mesh BG lure	15	65.2^c^	52.6–94.8
		4C	15	65.2^c^	48.5–87.6
		3C	15	68.4^c^	51.1–91.8

^†^ Number of mosquitoes collected was not significantly different by the Holm’s test (*P*> 0.05).

*Trap failures caused unbalanced trap nights.

In Virginia, the number of adult *Ae*. *albopictus* collected from BGS2P with white cloth was significantly different based on the lure type (χ^2^ = 57.6, *P*< 0.0001). BGS2P with white cloth baited with mesh BG lure, 4C, and 3C, each collected significantly higher numbers of adults (Z = 5.03, *P*<0.0001; Z = 4.66, *P*<0.0001; Z = 4.88, *P*<0.0001) compared to the 1C and no lure. 1C collected significantly higher numbers compared to traps with no lure (Table 2, Z = 2.69, *P* < 0.02).

### Experiment 3: Addition of white cloths to BGS2P

For BGS2P and BGS2P with white cloth, a total of 152 *Ae*. *albopictus* adults (123 females and 29 males) were collected in Louisiana, and 838 *Ae*. *albopictus* adults (603 females and 235 males) were collected in New Jersey. No significant differences in the number of adult *Ae*. *albopictus* collected using either BGS2P or BGS2P with white cloth for any of the trap configurations were detected (Table 3). A total of 10,324 *Ae*. *albopictus* adults (7,649 females and 2,675 males) were collected using BGS1 and BGS2P with and without a white cloth in Virginia. The BGS1 outperformed both BGS2P and BGS2P with white cloth (Z = 5.87, *P*<0.0001), while there were no significant differences between the BGS2P traps themselves.

**Table 3:** Comparison of the number of adult *Ae*. *albopictus* collected in New Jersey and Louisiana, USA, using BGS2P and BGS2P with white cloth with and without lures.

Location	Trap type	Lure type	No. of trap nights	LS mean no. of *Ae*. *albopictus* †	95% CI
**New Jersey**	BGS2P	No lure	22	10.2	6.9–15.3
		4C	23	8	5.4–11.9
	BGS2P with white cloth	No lure	23	6.3	4.2–9.4
		4C	23	9	6.0–13.3
**Louisiana**	BGS2P	4C	21	3.59	2.5–5.2
	BGS2P with white cloth	4C	21	3.63	2.5–5.3
**Virginia**	BGS1	Mesh BG lure+CO_2_	18	162.0^a^	134.8–194.5
	BGS2P	Mesh BG lure+CO_2_	18	73.3^b^	60.6–88.6
	BGS2P with white cloth	Mesh BG lure+CO_2_	18	66.0^bc^	54.5–79.9

^†^ Number of mosquitoes collected within each location was not significantly different by the Holm’s test (*P* > 0.05).

### Experiment 4: Comparisons between various lures using BGS1 and BGS2

A total of 382 *Ae*. *albopictus* adults (299 females and 83 males) were collected using the BGS1 and BGS2P in Florida. We did not have enough data to analyze BGS1 and BGS2P with no lure. BGS1 with mesh BG lure collected significantly higher adult numbers compared to BGS2P with mesh BG lure (χ^2^ = 13.8, df = 1, *P*< 0.0002). BGS1 with mesh BG lure, CO_2_ and octenol collected significantly higher adult numbers compared to BGS2P with mesh BG lure, CO_2_ and octenol (χ^2^ = 82.7, df = 1, *P*< 0.0001) (Table 4).

**Table 4:** Comparison of the number of adult *Ae*. *albopictus* collected using BGS1 and BGS2P traps with and without lures in Florida, USA.

Trap type	Lure type	No. of trap nights	Mean no. of *Ae*. *albopictus*	95% CI
**BGS1**	No lure	3	1.3	0.68–2.94
**BGS2P**	No lure		1.6	0.4–4.4
**BGS1**	Mesh BG lure	3	18.6	10.1–27.2
**BGS2P**	Mesh BG lure		7.6	4.8–16.4
**BGS1**	BG lure cartridge+CO2+octenol	3	75	27.4–122.5
**BGS2P**	BG lure cartridge+CO2+octenol		23	14.1–49.6

## Discussion

Adult mosquito surveillance is a fundamental component of integrated mosquito management programs tasked with preserving the quality of life and protecting the public health of their constituents. Surveillance data is used to identify the location and magnitude of mosquito populations and to determine the field infection rates as well as the transmission potential for mosquito-borne diseases. More importantly, surveillance data may be used to gauge the efficiency and efficacy of mosquito control operations, with the larger aim of reducing economic costs and environmental impacts. Thus, it is imperative to develop and utilize tools that may be reliably and effectively used for the accurate and consistent surveillance of targeted mosquito populations. The development and commercial availability of the BGS trap provided a much needed surveillance tool to monitor increasingly expanding populations of the invasive *Ae*. *aegypti* and *Ae*. *albopictus* globally. Because of the high capture rates and specificity of the trap for these species, the BGS trap has quickly become the golden standard tool used for *Aedes* surveillance [6,7]. However, continuing functionality problems and inconsistencies during field use limited the utility and reliability of this trap for researchers and mosquito control personnel [14]. The need to reduce construction defects and increase the field durability of the trap led to the creation of the next generation of BGS traps. Our studies evaluated the capture efficiency of the newly designed PVC-coated BGS2P trap, with and without a variety of lures, against the original BGS1. Our assessments were made across a large geographic area, allowing for a broad sampling of *Ae*. *albopictus* throughout much of its invasive range in temperate North America. Overall, we found that the BGS1 trap collected two to three times more *Ae*. *albopictus* than the prototype trap in Florida, Louisiana, New Jersey, and Virginia, regardless of the type of lure or CO_2_ used for the investigations. A previous study has also shown a two-fold increase in the collection of *Ae*. *albopictus* using the BGS1 trap type versus the BGS2 prototype [17]. Additionally, these authors have also shown that the BGS1, without the addition of any attractants, is capable of collecting 2.3 times more *Ae*. *albopictus* than the BGS2P with no attractants [17]. Our investigations in Louisiana and New Jersey also captured 2.2 and 2.3 times, respectively, more *Ae*. *albopictus* in the BGS1 trap with no attractants than the BGS2P with no attractants. Clearly the visual attractiveness of the BGS1 trap must be recognized in comparison to the BGS2P, supporting previous studies suggesting the importance of visual cues for collection of *Ae*. *albopictus* field populations [7,17].

The addition of the mesh BG lure or new BG cartridge lure component to the BGS2P significantly increased the capture rates for *Ae*. *albopictus* in Virginia, although the addition of those lures did not affect the number of adults collected in New Jersey. The addition of BG lures significantly increased catch counts when compared to the 1C and no lure. These results are not in agreement with Arimoto et al. (2015) which found that the 3-component cartridge lure collected nearly 1.5 times more *Ae*. *albopictus* than the standard mesh BG lure [17]. Lastly, the addition of the white cloth in BGS2P did not increase *Ae*. *albopictus* collections, supporting statements made by Arimoto et al. (2015) that the textile used for the BGS2P trap body may have repelled mosquitoes.

Carbon dioxide is considered a universal attractant for hematophagous insects, especially mosquitoes[18]and has been used extensively to enhance capture rates in many field studies [9,19–21]. Used singly or in combination, various lures have also resulted in varying effects for different trap types [7,19,22]. Our study is in agreement with previous studies showing CO_2_ in combination with other lures increases *Ae*. *albopictus* collections for BGS1 and BGS2P.

The BGS2 prototype used during these studies required no maintenance or replacement parts, due in part to an upgraded design which includes a self-supporting structure, PVC-coated canvas trap body, and air actuated flap over the intake pipe. However, reduced performance was observed. As reported by Arimoto et al. (2015), there is a possibility of the trap body textile having a repelling affect [17]. The balance between a sturdier material (blue PVC-coated canvas is sturdier than the PE non-woven fabric material) and performance is needed for a new version of BGS traps. Based on our results and the results of Arimoto et al. (2016), Biogents AG has replaced the trap body material from PVC coated canvas material to the original PE non-woven fabric material in the commercially available BG Sentinel 2 traps with. Biogents AG has retained the original trap body material for the newly designed BGS2; however, the color of the new trap is now a darker blue, instead of the white coloration on the original BGS1.

With the population increase and geographic expansion of *Aedes* mosquitoes and the emergence of exotic arboviruses that they vector, such as chikungunya and Zika, a growing need exists for accurate surveillance tools. Additionally, increasing vector control options, such as genetic control methods in the form of sterile insect techniques or manipulations using *Wolbachia*, require accurate assessment of field populations [23,24]. The success of these novel control methods will depend largely on surveillance data to gain knowledge of the survival, dispersal, and the longevity of targeted mosquitoes. Continued modifications and field evaluations of BGS traps will provide essential surveillance data for public health stewards charged with the difficult task of keeping invasive mosquitoes and the pathogens that they transmit at bay. It is our expectation that a balance will be reached which combines field durability and longevity of the traps, in conjunction with efficacy, for sustainable field surveillance of *Aedes* species.

## Supporting information

S1 Biogents trap comparison data (XLSX)
